# *In planta* Activity of the Novel Copper Product HA + Cu(II) Based on a Biocompatible Drug Delivery System on Vine Physiology and Trials for the Control of Botryosphaeria Dieback

**DOI:** 10.3389/fpls.2021.693995

**Published:** 2021-09-03

**Authors:** Vincenzo Mondello, Olivier Fernandez, Jean-François Guise, Patricia Trotel-Aziz, Florence Fontaine

**Affiliations:** Université de Reims Champagne-Ardenne, Unité Résistance Induite et Bioprotection des Plantes RIBP EA 4707, USC INRAE 1488, SFR Condorcet CNRS 3417, BP1039, Reims, France

**Keywords:** Botryosphaeria dieback, chemical control, copper, elicitation, hydroxyapatite, plant response, *Vitis vinifera*

## Abstract

The growing concerns on human and environment health are forcing the plant protection industry toward the formulation of more eco-sustainable plant protection products (PPP), both efficient and innovative in their approach to disease control. A large number of these innovative formulations now rely on a combination of pathogens antagonistic properties and stimulation of natural plant defense to pathogens. The formulation HA + Cu(II), in which copper is delivered to the plants by the drug-delivery molecule hydroxyapatite (HA), was found efficient against the grapevine pathogens *Plasmopara viticola* and *Phaeoacremonium minimum* and able to induce the host-plant defense system. We investigated the HA + Cu(II) impacts on grapevine physiology, both in uninfected and when infected by the Botryosphaeria dieback agents *Diplodia seriata* and *Neofusicoccum parvum*. This study of plant physiology and disease impact were addressed to evaluate both the HA + Cu(II) potential as a plant defense elicitor and its possible and future use as PPP in vineyard. Our results showed that HA + Cu(II) induced several key-defense genes without negatively affecting plant growth and photosynthetic activity. In addition, fungistatic effect on the two *Botryosphaeriaceae* at the *in planta* tested concentrations is reported. Altogether, our results obtained under controlled conditions fully support the potential of HA + Cu(II) as a promising PPP toward grapevine trunk diseases in vineyard.

## Introduction

The acronym GTDs (grapevine trunk diseases) encompass a group of destructive fungal diseases that attack and colonize the wood of perennial organs of grapevine, resulting in grape production loss, progressive decline and ultimately plant death (Mugnai et al., 1999; Úrbez-Torres, 2011; Bertsch et al., 2013). Compared to other GTDs, Botryosphaeria dieback has been fully described recently (Úrbez-Torres, 2011), although its first report under the designation “Black dead arm” date back to almost fifty years earlier (Lehoczky, 1974). The various fungal genera associated with this disease, *Botryosphaeriaceae*, are also well-known pathogens of other crops and forest plants (Giambra et al., 2016; Aćimović et al., 2018; Elena et al., 2018; Bettenfeld et al., 2020; Hilário et al., 2020). The increasing negative economic impact of GTDs worldwide, both in vineyards and nurseries (Fontaine et al., 2016), has attracted the attention of winegrowers, scientists, and chemical companies committed to safeguarding the viticulture industry. Despite years of research into the management of GTDs, we still lack simple and efficient control methods (Mondello et al., 2018a). Several active ingredients (AIs) formerly used to control GTDs (e.g., sodium arsenite, benomyl, and carbendazim; for review Mondello et al., 2018b) are no longer allowed for use in Europe.

As reported by Armengol (2014) and reviewed by Gramaje et al. (2018), the successful management of GTDs requires a transversal strategy along the productive life cycle of grapevine, from the nursery to the vineyard. However, because of growing public concern, GTD control must be done sustainably, especially because viticulture is still highly depending on pesticides (Zucca et al., 2009).

Several options are currently being tested. A first one uses grape cultivars resistant or tolerant to diseases. Unfortunately, to date and unlike for other major grapevine diseases such as downy and powdery mildew, no source of genetic tolerance to GTDs has been found in the *Vitis vinifera* genome (Vezzulli et al., 2018). A second option is to use our knowledge of ecological and symbiotic interactions to develop biocontrol agents and bio-based compounds to control these diseases. Some of these compounds or agents are currently applied in organic viticulture (Mondello et al., 2018b). Due to the adherence to traditional cultivars and crop system management, mainly for economic reasons (Pertot et al., 2017), the integrated pest management (IPM), which “*emphasizes the growth of a healthy crop with the least possible disruption to agro-ecosystems and encourages natural pest control mechanisms*” (FAO definition), is still the primary strategy for achieving sustainability in viticulture. Therefore, the agrichemical industry is now focusing on the synthesis of new AIs that combine low ecotoxicological profiles with sustained efficacy.

Copper is one of the most common fungicidal AIs in vineyards and the only one available for use in organic viticulture to control foliar diseases such as downy mildew (Berkelmann-Löhnertz et al., 2012). On the one hand, copper-based products offer a broad spectrum of activity toward oomycetes, fungi and bacteria. On the other hand, cupric substances, because of their potential for phytotoxic effects and the risk of accumulation in vineyards soil and water (Rusjan et al., 2007; Brunetto et al., 2014), are now considered by the European Community (EC) as “candidates for substitution” (art. 24 EC Regulation n. 1107/2009). Indeed, European legislative interventions have been regularly issued to progressively reduce the use of cupric fungicides in agriculture. Currently, the limit is fixed at a maximum of 28 kg/ha of metallic copper within 7 years, or 4 kg/ha/year (1981/2018 EC regulation).

Nevertheless, copper-based fungicides are not easily replaceable and still widely applied in vineyards around the world, especially to control the emergence of fungicide-resistant strains of *Plasmopara viticola*, the causal agent of downy mildew. Many strategies to reduce the copper content of pesticides are currently under investigation, namely (i) reducing its particle size (micronization), (ii) regulating its release and resistance to rain (encapsulation), and (iii) combining it with other substances (e.g., zeolites, clay, terpene alcohol) to regulate copper bioavailability according to environmental conditions (La Torre et al., 2018). HA + Cu(II), tested in the present study, belongs to this last group. In this formulation, copper sulfate pentahydrate salt (Cu^2+^ 35 g/L) is vehiculated throughout the plant thanks to the carrier molecule Hydroxyapatite (Roveri et al., 2016). Against *Phaeoacremonium minimum*, an Esca complex pathogen on grapevine, HA + Cu(II) limits this pathogen both *in vitro* and in the nursery when applied during the misting of cuttings (Battiston et al., 2018). Work by Battiston et al. (2018, 2019, 2021), beside the ability of HA + Cu(II) to control both *Botrytis cinerea in vitro* and *P. viticola* on grapevine cuttings under greenhouse conditions, has also assessed the elicitation properties of this formulation toward genes linked to the host-plant defense system.

The aims of this study were to evaluate the efficiency of HA + Cu(II) as plant defense elicitor in grapevine and to verify its possible use as plant protection products (PPP) against two GTD pathogens belonging to the family of *Botryosphaeriaceae*. Two independent bioassays were thus conducted to analyze the effects of HA + Cu(II) during Botryosphaeria dieback disease progression (*in planta*) on (i) vine physiology (plant growth and photosynthetic activity), (ii) defense responses (induction of defense gene expression), and (iii) on pathogen growth and survival (*in vitro* and *in planta*). All the *in planta* experiments were carried out on cuttings of “Chardonnay” and “Cabernet sauvignon” cultivars, both showing different susceptibility to GTDs.

## Materials and Methods

### Plant Material and Fungal Strains

The effects of HA + Cu(II) formulation, namely LC2017 (Natural Development Group, Castel Maggiore, Italy), on host-plant defenses elicitation and on vine physiology were assessed under controlled conditions using the two *V. vinifera* cultivars “Chardonnay” and “Cabernet sauvignon.” These two cultivars, harboring different susceptibility to *Botryosphaeriaceae*, with “Chardonnay” being more tolerant compared to “Cabernet sauvignon” (Bruez et al., 2013; Fontaine et al., 2016; Guerin-Dubrana et al., 2019), were chosen to evaluate the potential use of LC2017 as PPP for the control of these pathogens. The method of vegetative cuttings, as described in Reis et al. (2019), was used here as artificial inoculation assay.

Two strains of *Diplodia seriata*, namely Ds 98-1 and Ds 99-7 (Reis et al., 2016), and of *Neofusicoccum parvum*, namely Np bour (Ramírez-Suero et al., 2014) and Np bt67 (Trotel-Aziz et al., 2019), were used in the artificial inoculations. These two fungal species, among those associated with Botryosphaeria dieback, differ in their aggressiveness toward *V. vinifera*, with *N. parvum* considered the more virulent (Úrbez-Torres and Gubler, 2009; Pitt et al., 2013).

### Production of Vegetative Cuttings and Artificial Infection

**FIGURE 1:**
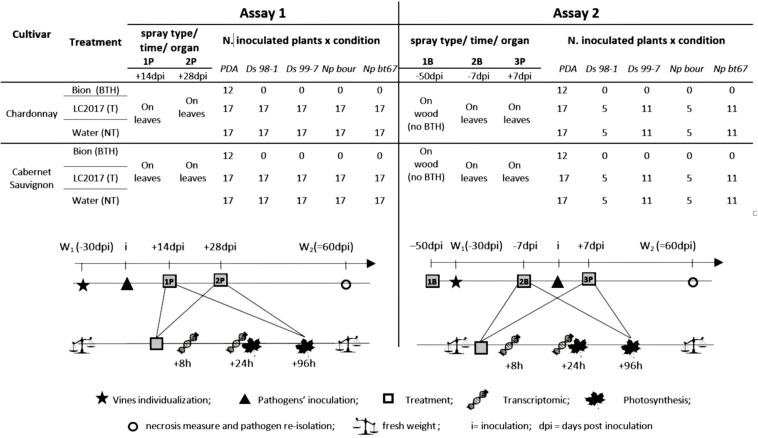
Scheme of the greenhouse bioassays followed to evaluate the effects of LC2017 on vine physiology and Botryosphaeria dieback development. For each assay are reported the different treatments (BTH = treated with commercial product elicitor of plant defense system; T = treated with LC2017; NT = treated with water), the number and the type (P = Post inoculation, B = Before inoculation) and their timing according to artificial inoculations (dpi = days post inoculation), the target organ of treatment and the number of vines followed for each condition. Below, the timing followed for pathogens inoculation (i), treatments, photosynthesis measures, transcriptomic study, plant fresh weight measures, necroses measure, and pathogen re-isolation.

Three-node cuttings of “Chardonnay” and “Cabernet sauvignon” (about 500 per cultivar in Assay 1 and likewise 260 in Assay 2) were first disinfected, by dipping them into a 0.05% 8-Hydroxy-chinolin-sulfat solution (4 h at 28°C), then dried, waxed in the upper part, and stored at 4°C for at least 40 days. After this cold storage, the cuttings were hydrated overnight in a 0.05% 8-hydroxy-chinolin-sulfat bath, at 28°C. Once dry, the cuttings were transferred into the greenhouse and their first and second nodes disbudded, with the basal cuts refreshed with scissors, and immersed for 30 s in an indole-3-butyric acid (AIB) solution (1 mg/mL). These AIB–treated cuttings were put into plastic pots (20 cuttings/pot) that containing horticulture soil (Sorexto M4600) and placed under controlled conditions - 24°C, 80% relative humidity (R.H.), and a photoperiod of 16-h/8-h light/dark for rooting. When the green shoots had 5 or 6 leaves (3-week-old), the vegetative cuttings were gently uprooted, checked for the presence of a well-developed root system, and singly transferred to 0.5 L pots. At this stage (W_1_ in Figure 1), to evaluate the effect of the treatments on vines’ whole fresh weight, 50 plants of each cultivar [5 replications × 5 conditions × 2 experiments: LC2017 (T) and water (NT)] were gently rinsed under tap water to eliminate the soil from their roots, then quickly dried on paper sheets, and finally weighed and marked. Rooted cuttings in single pots were left under the controlled conditions as previously described (24°C, 80% R.H., photoperiod of 16 h/8 h). Fifteen and thirty days since their transplantation into single pots, each vine was fertilized with 30 mL of a nutritive solution (Lesaint media; Coïc and Lesaint, 1983) containing macro- and micro-nutrients.

In both bioassays, to evaluate the LC2017 effects as PPP against Botryosphaeria dieback pathogens, after 30 days of growth in single pot, some plants were artificially inoculated with *D. seriata* (Ds 98-1 and Ds 99-7) or *N. parvum* (Np bt67 and Np bour) strains, according to Reis et al. (2016, 2019) and Trotel-Aziz et al. (2019) and kept in similar greenhouse conditions.

### Greenhouse Assays: Experimental Design and LC2017 Treatments

In the greenhouse, two independent experimental bioassays (Assay 1 and Assay 2) were performed, as conveyed in Figure 1. In Assay 1, to evaluate the LC2017 elicitor potential and its effects on vine’s physiology, an aqueous solution of LC2017 at 0.5% v/v (in distilled H_2_O) was sprayed twice on the leaves, both adaxial and abaxial sides, to completely cover the leaf without any dripping (Supplementary Figure 1A). To analyze the effect of LC2017 in a possible control strategy against Botryosphaeria dieback, the two sprays with LC2017 were done, respectively, at 14 (1P = first Post-inoculation treatment) and 28 (2P = second Post-inoculation treatment) day post inoculation (dpi) of the *Botryosphaeriaceae* strains. Contemporary, distilled water (NT) and BTH [S-methyl benzo (1,2,3) thiadiazole-7-carbothioate: 150 mg of the commercial product BION^®^, (Syngenta- France) in 1 L of distilled water] were, respectively, applied as the negative and positive controls for the transcriptomic analysis. More precisely, since BTH is a well-documented elicitor of plant defense (Uknes et al., 1996) currently available on the market, it was chosen as an “elicitor” control. Finally, three vines were used to measure photosynthetic activity at 24 and 96 h after each treatment.

The second assay (Assay 2) was done firstly to confirm the LC2017 effect on vine’s physiology and secondly to test a second Botryosphaeria dieback control strategy, by a different timing and number of applications (Figure 1): three LC2017 sprays were applied, two before the pathogens’ inoculation, at −50 (1B = first treatment Before-inoculation) and −7 dpi (2B + second treatment Before-inoculation), and one after the inoculation, at +7 dpi (3P = third treatment, Post-inoculation treatment). At 1B, LC2017 (0.5%) and water (NT) were sprayed onto the bark and in the wound of rooted cuttings, after simulating a typical wound of winter pruning (Supplementary Figures 1B,C). No treatment with BTH was performed at this time-point. At B2 and P3, LC2017 was sprayed at 0.5% on each leaf side, as done in Assay 1; likewise and at the same times, distilled water (NT) and BTH were applied.

Similar to Assay 1, all four pathogenic strains were used to repeat the monitoring of the effect of LC2017 on vine’s physiology and disease progression, replacing the *N. parvum* Np bour strain with Np bt67 one for transcriptomic investigation. Following Assay 1 results, photosynthetic activity was measured for non-inoculated vines and, among those inoculated, only for Ds 99-7- and Np bt67-inoculated plants by using 3 out of 5 vines employed for evaluating the effect of LC2017 on plant whole fresh weight.

### Gene Expression by Targeted RT-qPCR

To decipher the potential elicitor effects of LC2017 on vine physiology, a gene expression analysis was set up. The leaf sampling for this transcript analysis was performed in Assay 1 for NT, T, BTH and, among those inoculated, for T and NT vines infected by Ds 99-7 and Np bour at 8 h and 24 h after the last treatment (2P) and at 8 h after the second (2B) and third (3P) treatment in Assay 2 (as indicated in Figure 1). Each sample consisted of pooling the two leaves immediately above and below the inoculation point, with three plants per condition being used as replication for each time-point. Leaves collected were immediately frozen in liquid nitrogen and stored at −80°C. After each sampling time, the vines from which leaves were collected were discarded and no longer studied. Following the RNA extractions, quantitative real-time RT-PCR analysis was carried out according to Magnin-Robert et al. (2014) and Spagnolo et al. (2017), respectively. Results were expressed as the values of relative expression levels (ΔΔCt), corresponding to the mean of three independent biological replicates. To normalize genes expression level, 39SRP, 60SRP and EF-1α genes were used.

The specific primers for the targeted genes are listed in Supplementary Table 1. These genes were selected based on similar previous studies (Magnin-Robert et al., 2011, 2014, 2016; Spagnolo et al., 2014, 2017; Trotel-Aziz et al., 2019). Specifically, 13 genes were chosen to evaluate the grapevine cultivars response to LC2017 treatments. Among these, those linked to the phenylpropanoid pathway (*PAL* and *STS*) and other defense protein markers (*CHIT4C*, *GLUC, PR1*, and *PR10*), to detoxification processes (*GTS1*), to photosynthetic activity (*PsbP1* and *RbcL*), and to the “recovered” health-status markers highlighted in leaves of GTD-infected vines treated with sodium arsenite (*PME25, MSR, WRKY*, and *Hyd2*; Fontaine F., personal communication). The genes analyzed were considered to be up- or down-regulated when changes in their expression were either >2-fold or <0.5-fold. Furthermore, data were submitted to statistical analysis.

### Effect of LC2017 on Vine Physiology

#### LC2017 Treatments’ Effect on Plants’ Fresh Weight

In both assays, the whole fresh weight of five plants per condition was measured at the beginning (W1) and at 60 dpi (W2) (Figure 1). The ratio (W2–W1)/W1 (weight-fold increase) was calculated to estimate the impact of LC2017 upon plant growth.

#### Photosynthesis Measurement

The effect of LC2017 treatments on photosynthetic activity was measured using a portable infrared gas analysis system (LI-COR Model 6400-XT, Lincoln, NE, United States). Here, photosynthetic activity was measured by fixing the photosynthetically active radiation (PAR) intensity to 750 μmol of photons/cm^2^/s, the CO_2_ flux to 400 mMol/min, and the relative humidity to 50%.

In both assays, three vines per condition underwent LI-COR measurements at 24 h and 96 h after the LC2017 treatments (1P and 2P in Assay 1; B2 and P3 in Assay 2), to analyze its short- and long-term effects on photosynthetic activity. In Assay 2, due to the similarity between Pn values of vines inoculated with the same species, we measured only photosynthetic activity of Ds 99-7 and Np bt67 inoculated vines. BTH-treated vines were not measured for photosynthesis, not being an aim of the study. For each time-point, the following were measured: net photosynthesis (Pn, in μmol CO_2_ m^–2^s^–1^), stomatal conductance (G_s,_ in mol H_2_O m^–2^s^–1^), intercellular CO_2_ (Ci, in μmol CO_2_ mol^–1^) and transpiration (Tr, in mmol H_2_O m^–2^s^–1^).

### Effect of LC2017 on Botryosphaeria Dieback

#### *In vitro* Evaluation of the Fungistatic or Fungicidal Effect of LC2017 Against *Botryosphaeriaceae*

The activity of LC2017 toward *Botryosphaeriaceae* was first evaluated *in vitro* by growing the four strains on potato dextrose agar (PDA) plates enriched with different concentrations of the product. LC2017 was added to the autoclaved liquid medium (after cooling at 55°C) to achieve concentrations of 0% (i.e., the control), 0.25%, 0.5%, 1.0%, and 1.5%. Plates were then inoculated with a 3-mm mycelial plug taken from a 1-week-old pathogen and incubated at 28°C in the dark. Pathogen growth was calculated daily, by measuring two orthogonal diameters, through 9 dpi. At the end of each test, to assess the fungicidal or fungistatic activity of LC2017, those mycelial plugs that did not show any development after 9 dpi were transferred to new PDA plates deprived of LC2017; these were incubated at 28°C to observe their potential growth recovery over a period of 7 consecutive days. Each experimental treatment was replicated three times.

### Effect of LC2017 on Botryosphaeria Dieback Development *in planta*: Necrosis Length Measurements and Pathogen Re-isolation

The evaluation of the LC2017 effect on the progression of Botryosphaeria dieback was carried out in each assay at 60 dpi (W2 in Figure 1), with the same replicates (5) also used to calculate the plants’ whole fresh weight. The internal lesion length and pathogen re-isolation frequencies (IF) were both considered. For this purpose, and operating in a sterile laminar flow chamber, the cuttings’ area surrounding each inoculation point was first disinfected with 70% ethanol and rapid flame passages, to access and photograph the internal lesion size and remove from its thin wood slices with a sterile scalpel. To measure each lesion’s length, width in cm, and surface area in cm^2^, lesion pictures were analyzed in the free software program, ImageJ.^1^ Meanwhile, the small wood pieces taken from the necrosis edges were placed in Petri dishes containing PDA enriched with 100 mg/L streptomycin, to prevent bacterial contamination while waiting for the pathogen to undergo its growth recovery. The inoculated plates were kept at 28°C and observed over 7 days. The ratio “number of *Botryosphaeriaceae*-like mycelium growth/number of wood pieces” was used to calculate the relative IF.

### Statistical Analysis

All the data collected in the described experiments were analyzed with Mann-Whitney non-parametric test in GraphPad Prism v.5.0 (GraphPad Software, San Diego, CA, United States^2^).

## Results

### Effect of Treatments on the Expression Levels of Plant Defense-Related Genes

#### LC2017 and BTH Elicitation Effect in Non-inoculated Vines

**FIGURE 2:**
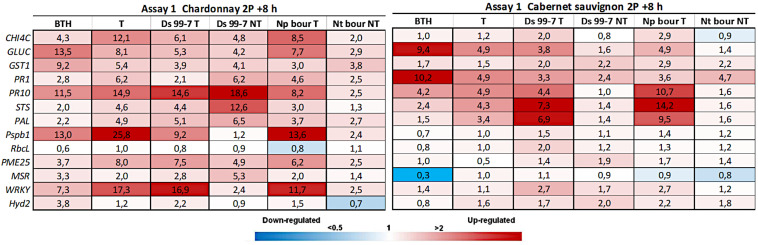
Expression levels of the selected 13 genes recorded by RT-qPCR in “Chardonnay” and “Cabernet sauvignon” 8h after the second LC2017 treatment (2P) in Assay 1. Values (mean of three technical replicates) represent the expression levels (ΔΔCt) of reported conditions relatively to the control (NT). Expression of a given gene was considered up- or down-regulated when value of relative expression was >2-fold or <0.5-fold compared to the control, respectively.

In Assay 1, 8 h after the second treatment (2P = 28 dpi) both BTH and LC2017 induced in “Chardonnay” and “Cabernet sauvignon” several defense-marker genes, with higher values in LC2017 treated plants (Figure 2). Statistical analysis often showed significance for the same genes in the BTH and T conditions, with few differences according to the considered cultivar or P value (Table 1) when compared to the control (NT). Gene expression data recorded 24 h after treatment with LC2017, showed no induction effect for both cultivars, as well as for BTH (*data not shown*).

**TABLE 1:**
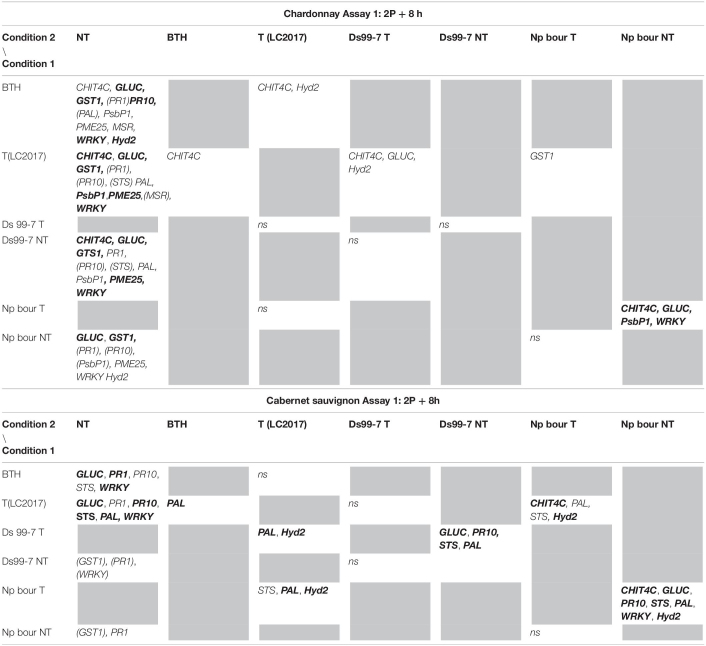
Results of the statistical analysis (Mann-Whitney *U* test) on Assay 1 transcriptomic data recorded in vines “Chardonnay” (upper part) and “Cabernet sauvignon” (lower part) at 2P + 8 h: the reported genes resulted with both relative expression >2 and significantly induced for *p* < 0.05 (in **bold** those significant for *p* < 0.01) in condition 1 when compared to the other conditions (condition 2).

*In brackets, genes resulted with relative expression >2 but not significant in Mann-Whitney U test. In gray, the comparison not analyzed; ns indicate no statistical differences among the targeted genes.*

**FIGURE 3:**
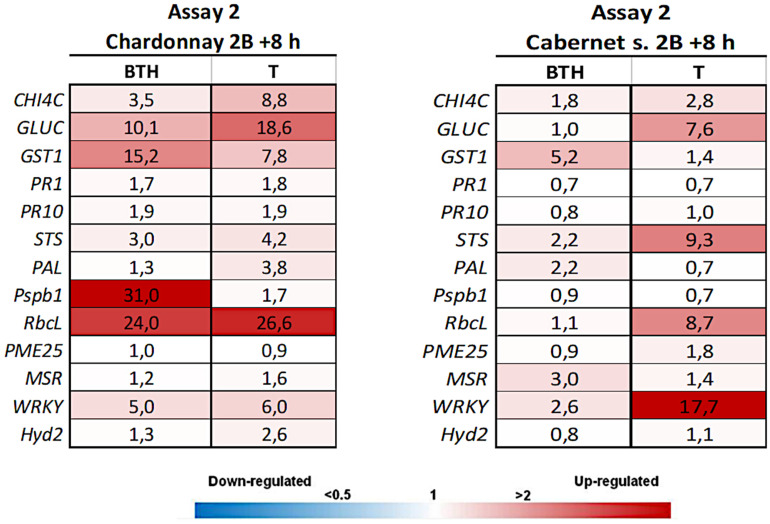
Expression levels of the selected 13 genes recorded by RT-qPCR in “Chardonnay” and “Cabernet sauvignon” 8 h after the second LC2017 treatment (2B) in Assay 2. Values (mean of three technical replicates) represent the expression levels (ΔΔCt) of reported conditions relatively to the control (NT). Expression of a given gene was considered up- or down-regulated when value of relative expression was >2-fold or <0.5-fold compared to the control, respectively.

**FIGURE 4:**
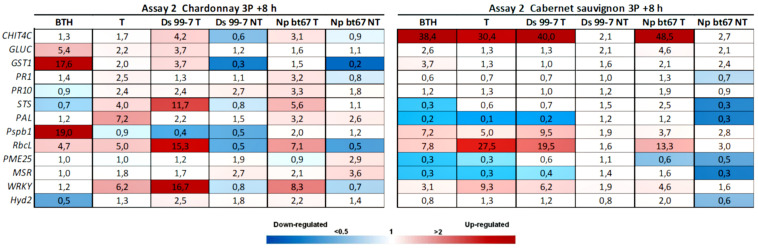
Expression levels of the selected 13 genes recorded by RT-qPCR in “Chardonnay” and “Cabernet sauvignon” 8 h after the third LC2017 treatment (3P) in Assay 2. Values (mean of three technical replicates) represent the expression levels (ΔΔCt) of reported conditions relatively to the control (NT). Expression of a given gene was considered up- or down-regulated when value of relative expression was >2-fold or <0.5-fold compared to the control, respectively.

In Assay 2, the second LC2017 treatment (2B) elicited, with few exceptions, the same genes as BTH in “Chardonnay” with higher induction values, while on “Cabernet sauvignon” genes were differently elicited according to the treatment (Figure 3). The LC2017’s capability at enhancing the defense responses was confirmed in the following treatment on “Chardonnay” (3P): even with lower values, the LC2017 induced more genes than BTH did (Figure 4). On the contrary, in “Cabernet sauvignon” both BTH and LC2017 showed a lower induction effect, also determining the repression of some genes (*PAL, PME25*, and *MSR*). Differently to Assay 1, the overexpression of some genes resulted not significant for BTH and LC2017 in both cultivars (Table 2).

**TABLE 2:**
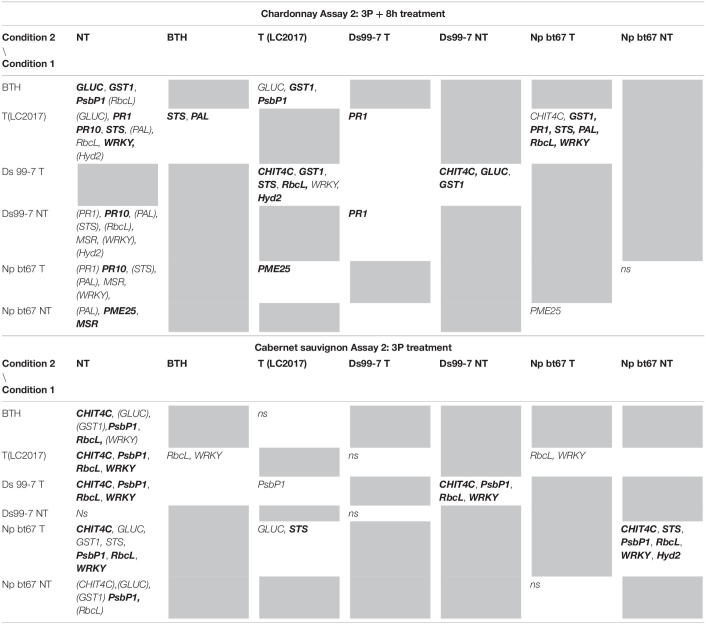
Results of the statistical analysis (Mann-Whitney *U* test) on Assay 1 transcriptomic data recorded in vines “Chardonnay” (upper part) and “Cabernet sauvignon” (lower part) at 3P + 8 h: the reported genes resulted with both relative expression >2 and significantly induced for *p* < 0.05 (in **bold** those significant for *p* < 0.01) in condition 1 when compared to the other conditions (condition 2).

*In brackets, genes resulted with relative expression >2 but not significant in Mann-Whitney U test. In gray, the comparison not analyzed; ns indicate no statistical differences among the targeted genes.*

#### Pathogen Elicitation Effect in Not Treated Vines

In Assay 1, the pathogens presence induced the plant defense system, especially in “Chardonnay,” where up to 10 genes resulted overexpressed (> 2 folds) by *D. seriata* and *N. parvum*, while few were overexpressed in “Cabernet sauvignon” (Figure 2). In Assay 2, in which the transcriptomic study was performed earlier than the one of Assay 1 (+ 7 dpi *vs* + 28 dpi) only few genes resulted overexpressed in both cultivars. On the contrary, several genes were repressed (i.e., *GST1* in “Chardonnay” Ds 99-7 NT) or close to the value limit of <0.5 (*RbcL* in “Chardonnay” Np bour NT) (Figure 4). Finally, gene expression data showed the effect of both pathogens *D. seriata* and *N. parvum* alone upon the expression of targeted genes, thereby highlighting differences specific to the strain and cultivar. In both years, in “Chardonnay,” up to eight and four genes were significantly induced by Ds 99-7 NT and Np bour NT, respectively (Tables 1, 2). These numbers were correspondingly reduced to two and three in “Cabernet sauvignon.”

#### LC2017 Elicitation Effect in Inoculated Vines

In Assay 1 LC2017 often increased the overexpression values recorded in NT vines in both cultivars, as in “Chardonnay” infected by Np bour and in Ds 99-7- or Np bour- infected “Cabernet sauvignon” in which, moreover, the treatment was able to induce several genes, not observed in infected and not treated plants (Figure 2). Statistical analysis on overexpressed genes values between T and NT conditions (Table 1) revealed significance for *CHIT4C, GLUC, and WRKY* in “Chardonnay” and *CHIT4C, GLUC, PR10, STS, PAL, WRKY*, and *Hyd2* in “Cabernet sauvignon” treated and infected by *N. parvum.* In Ds 99-7*-*treated plants, significant inductions were observed only in “Cabernet sauvignon” for *GLUC, PR10, STS, and PAL*, while those in “Chardonnay” were not.

Similarly to Assay 1, in presence of pathogen, LC2017 treatments performed in Assay 2 led to a higher induction of some genes in the inoculated vines of “Chardonnay” when compared to the non-treated ones. In “Cabernet sauvignon,” the LC2017 treatment blocked the gene repression recorded in the related non-treated condition (i.e., *STS, PAL, MSR* – Figure 4). According to the statistical analysis results (Table 2), the induction of *CHIT4C, GLUC*, and *GST1* in “Chardonnay” was significantly higher in Ds 99-7 vines in the T than NT condition, a result not observed in the first assay (*see* Table 1). No significant differences were observed in Np bt67-infected plants. On “Cabernet sauvignon,” the treatment significantly induced *CHIT4C*, *STS*, *PsbP1*, *RbcL*, *WRKY*, and *Hyd2* genes in Np bt67-inoculated vines. The genes *CHIT4C*, *STS*, and *WRKY* were thus confirmed as induced with statistical significance for *N. parvum* (Table 3). No LC2017 priming effects of the B1 and B2 treatments were observed in the targeted genes after inoculation with the pathogens.

**TABLE 3:** Kinetics of photosynthetic values (Pn, Gs and Tr) and main statistical parameters (median and standard deviation) recorded during Assay 2 one (+ 1d) and four (+ 4d) days after the second (2B) and third (3P) treatment on non-inoculated vines “Chardonnay” and “Cabernet sauvignon.”

	Chardonnay – Assay 2								Cabernet sauvignon – Assay 2							
	Control NT				Control T				Control NT				Control T			
	2B + 1 d	2B + 4 d	3P + 1 d	3P + 4 d	2B + 1 d	2B + 4 d	3P + 1 d	3P + 4 d	2B + 1 d	2B + 4 d	3P + 1 d	3P + 4 d	2B + 1 d	2B + 4 d	3P + 1 d	3P + 4 d
	**Net photosynthesis (Pn)**															

Median	10.26 **b**	5.36 **a**	5.79 **a**	5.32 **a**	8.59 **b**	3.08 **a**	3.80 **ab**	4.72 **ab**	0.45 **a**	4.12 **b**	2.55 **a**	5.31 **b**	3.56 **a**	7.96 **b**	5.81 **a**	4.72 **a**
SD	2.97	0.51	0.61	0.65	2.92	0.60	1.15	1.18	1.99	0.73	0.14	0.65	0.60	1.17	1.41	1.18

	**Stomatal conductance (Gs)**															

Median	0.13 **c**	0.05 **a**	0.09 **b**	0.08 **b**	0.09 **b**	0.03 **a**	0.06 **ab**	0.06 **ab**	0.04 **b**	0.00 **a**	0.02 **b**	0.08 **c**	0.13 **b**	0.03 **b**	0.04 **a**	0.07 **a**
SD	0.04	0.01	0.01	0.01	0.01	0.02	0.02	0.02	0.02	0.00	0.00	0.01	0.02	0.01	0.01	0.02

	**Intercellular CO_2_ (Ci)**															

Median	260.6 **b**	216.5 **a**	281.0 **c**	283.0 **b**	238.4 **a**	241.7 **a**	280.0 **a**	269. 7 **a**	368.1**d**	137.6 **a**	217.3 **b**	283.0 **c**	336.0 **d**	70.1 **a**	176.9 **b**	269.7 **c**
SD	4.3	21.5	9.2	28.6	36.1	30.5	12.5	7.2	113.5	13.0	22.3	28.5	11.0	9.2	9.01	7.19

	**Transpiration rate (Tr)**															

Median	1.89 **c**	0.92 **a**	1.53 **b**	1.42 **bc**	1.36 **b**	0.62 **a**	0.97 **ab**	1.14 **ab**	0.64 **a**	0.09 **a**	0.45 **a**	1.42 **b**	2.03 **d**	0.40 **a**	0.78 **b**	1.14 **bc**
SD	0.44	0.20	0.19	0.20	0.20	0.28	0.37	0.31	0.37	0.11	0.06	0.20	0.23	0.26	0.195	0.31

*2B = second treatment before infection; 3P = post-infection treatment. To different letters correspond statistical difference (p < 0.05) among the four kinetic point values of the same condition (Mann-Whitney U test).*

### Effect of LC2017 Treatments on Vine Physiology

#### Effect of LC2017 on Photosynthesis

Photosynthetic parameters recorded during Assay 1 are summarized in Tables 4, 5. Cultivars showed the same Pn values trend irrespective of T and NT condition considered: decreasing in “Chardonnay” with significant differences between the first and the last measurement, stable in “Cabernet sauvignon” without significant differences (Table 4). Overall, this decrease in Pn combined with the limited variation of the other photosynthetic parameters (G_s_, Ci, and Tr; Tables 4, 5) suggested a non-stomatal limitation had occurred. In presence of pathogens, treatments with LC2017 appeared to moderate or nullify the pathogen-induced reduction of Pn, with significant Pn values differences between NT and T conditions (Table 5). Details for the other the photosynthetic parameters measured in Assay 1 can be found in the Supplementary Tables 2–5.

**TABLE 4:** Evolution of photosynthetic parameters (Pn, Gs, Ci, and Tr) recorded during Assay 1 one (+ 1 d) and four (+ 4 d) days after each post infection treatment on non-inoculated grapevines of “Chardonnay” and “Cabernet sauvignon.”

	Chardonnay – Assay 1								Cabernet sauvignon – Assay 1							
	Control NT				Control T				Control NT				Control T			
	1P + 1d	1P + 4d	2P + 1d	2P + 4d	1P + 1d	1P + 4d	2P + 1d	2P + 4d	1P + 1d	1P + 4d	2P + 1d	2P + 4d	1P + 1d	1P + 4d	2P + 1d	2P + 4d
	**Net Photosynthesis (Pn)**															

Median	5.03 **b**	1.72 **ab**	2.55 **ab**	2.71 **a**	3.22 **b**	4.57 **b**	2.74 **ab**	0.80 **a**	3.42 **a**	2.50 **a**	3.57 **a**	1.94 **a**	3.00 **a**	3.00 **a**	3.44 **a**	3.13 **a**
SD	1.62	1.34	1.58	0.55	0.93	1.76	0.89	0.87	1.39	0.70	0.94	0.68	0.89	0.84	1.39	1.12

	**Stomatal conductance (Gs)**															

Median	0.12 **a**	0.06 **a**	0.04 **a**	0.04 **a**	0.07 **a**	0.10 **a**	0.07 **a**	0.07 **a**	0,07 **a**	0,05 **a**	0,09 **a**	0,05 **a**	0.06 **a**	0.06 **a**	0.04 **a**	0.03 **a**
SD	0.09	0.03	0.04	0.08	0.05	0.07	0.14	0.08	0,04	0,02	0,05	0,02	0.02	0.04	0.03	0.02

	**Intercellular CO_2_ (Ci)**															

Median	321.1 **b**	343.5 **b**	288.0 **a**	278.0 **ab**	316.0 **a**	283.5 **a**	358.3 **a**	375.8 **a**	307.5 **b**	285.8**a**	322.5**b**	337.6**c**	299.2**b**	312.4**ab**	261.0**ab**	214.4**a**
SD	15.2	8.8	38.9	46.7	11.1	89.1	34.3	98.0	113.5	13.0	22.3	28.5	8.3	32.9	21.1	55.1

	**Transpiration rate (Tr)**															

Median	1.79 **a**	0.86 **a**	0.63 **a**	0.68 **a**	1.03 **a**	1.60 **a**	1.07 **a**	0.38 **a**	1.28 **a**	0.72 **a**	1.46 **a**	0.89 **a**	1.12 **a**	1.17 **a**	0.68 **a**	0.57 **a**
SD	0.96	0.53	0.63	1.23	0.64	1.27	1.90	1.33	0.79	0.30	0.81	0.32	0.33	0.64	0.44	0.36

*1P = first treatment; 2P = second treatment. Data are the medians of three values ± SD. Different letters indicate significant differences (Mann-Whitney U test, p < 0.05) among the four time-point values within the same condition [T (treated) or NT (non-treated)].*

**TABLE 5:** Photosynthetic parameters (Pn and Ci) recorded during Assay 1 one (+ 1 d) and four (+ 4 d) days after each post infection treatment on grapevines “Chardonnay” and “Cabernet sauvignon” artificially inoculated with Botryosphaeria-dieback pathogens.

	Chardonnay – Assay 1								Cabernet sauvignon – Assay 1							
	Ds 98-1 NT				Ds 98-1 T				Ds98-1 NT				Ds 98-1 T			
	1P + 1d	1P + 4d	2P + 1d	2P + 4d	1P + 1d	1P + 4d	2P + 1d	2P + 4d	1P + 1d	1P + 4d	2P + 1d	2P + 4d	1P + 1d	1P + 4d	2P + 1d	2P + 4d
	**Net photosynthesis (Pn)**															

Median	3.68	2.00	1.86	−0.41	4.38	4.29*	2.29	1.93*	3.64	1.79	1.79	0.10	2.54	1.69	2.88	2.48*
SD	1.24	0.49	0.58	1.16	0.94	1.23	0.10	0.77	0.85	1.05	1.17	0.63	1.62	2.26	1.65	1.57
Median	356.57	346.69	292.9	292.94	338.7	315.0	359.1*	253.3*	326.9	316.8	207.8	379.4	250.15	304.52	313.25	197.8*
SD	15.9	42.7	46.6	62.7	6.7	29.8	10.2	47.2	32.1	24.4	164.3	37.5	44.8	35.5	68.00	145.1

	**Chardonnay – Assay 1**								**Cabernet sauvignon– Assay 1**							
	**Ds 99-7 NT**				**Ds 99-7 T**				**Ds 99-7 NT**				**Ds 99-7 T**			
	**1P + 1d**	**1P + 4d**	**2P + 1d**	**2P + 4d**	**1P + 1d**	**1P + 4d**	**2P + 1d**	**2P + 4d**	**1P + 1d**	**1P + 4d**	**2P + 1d**	**2P + 4d**	**1P + 1d**	**1P + 4d**	**2P + 1d**	**2P + 4d**

	**Net photosynthesis (Pn)**															

Median	2.18	−0.50	0.48	−0.09	3.35	1.80*	0.59	1.55*	2.79	1.36	2.09	0.44	3.01	2.56	2.80	2.73*
SD	1.30	1.18	0.63	0.69	0.80	0.74	1.46	0.27	0.72	0.90	0.23	0.85	0.70	0.82	1.32	0.67

	**Intercellular CO_2_ (Ci)**															

Median	267.6	456.5	374.0	392.3	310.2	244.0*	365.9	269.2*	302.0	280.6	173.7	361.6	284.7	240.7*	277.8	253.7*
SD	60.0	177.3	51.0	98. 6	20.9	127.5	1.3	61.2	17.0	10.7	76.2	35.3	24.7	32.5	59.9	28.4

	**Chardonnay– Assay 1**								**Cabernet sauvignon– Assay 1**							
	**Np bour NT**				**Np bour T**				**Np bour NT**				**Np bour T**			
	**1P + 1d**	**1P + 4d**	**2P + 1d**	**2P + 4d**	**1P + 1d**	**1P + 4d**	**2P + 1d**	**2P + 4d**	**1P + 1d**	**1P + 4d**	**2P + 1d**	**2P + 4d**	**1P + 1d**	**1P + 4d**	**2P + 1d**	**2P + 4d**

	**Net photosynthesis (Pn)**															

Median	1.82	4.36	1.75	1.57	2.51*	2.30	1.92	1.45	2.81	2.05	0.92	0.98	2.13	2.57	2.61	2.38*
SD	0.38	1.81	0.38	0.17	0.53	0.72	0.54	1.04	0.59	0.87	0.65	0.72	0.41	0.41	1.34	0.45

	**Intercellular CO_2_ (Ci)**															

Median	328.7	310.8	352.0	353.3	310.7	275.7	302.0	285.7*	339.2	333. 7	356.5	365.3	332.4	309.8	319.5	310.6*
SD	22.8	25.3	11.3	21.8	26.8	68.2	27.2	27.1	16.4	7.9	17.5	9.3	3.72	29.5	42.3	9.4

	**Chardonnay– Assay 1**								**Cabernet sauvignon– Assay 1**							
	**Np bt67 NT**				**Np bt67 T**				**Np bt67 NT**				**Np bt67 T**			
	**1P + 1d**	**1P + 4d**	**2P + 1d**	**2P + 4d**	**1P + 1d**	**1P + 4d**	**2P + 1d**	**2P + 4d**	**1P + 1d**	**1P + 4d**	**2P + 1d**	**2P + 4d**	**1P + 1d**	**1P + 4d**	**2P + 1d**	**2P + 4d**

	**Net photosynthesis (Pn)**															

Median	2.10	−1.12	0.54	0.35	1.56	2.52*	0.81	1.31	2.32	2.39	2.31	0.95	3.84	3.03	3.33	3.58*
SD	0.58	0.90	0.09	0.73	0.25	0.91	0.96	0.36	1.20	0.70	0.11	0.44	2.25	2.11	2.09	2.12

	**Intercellular CO_2_ (Ci)**															

Median	324.4	482.3	354.9	375.8	278.6	170.2*	364.8	254.9*	321.5	313.3	253.6	359.8	296.4	311.6	310.2	290.8*
SD	17.90	255.61	2.65	29.5	18.4	115.2	21.9	30.6	17.54	33.7	41.9	9.9	176.6	189.7	188.4	170.4
	**Chardonnay– Assay 1**								**Cabernet sauvignon– Assay 1**							
	**Np bt67 NT**				**Np bt67 T**				**Np bt67 NT**				**Np bt67 T**			
	**1P + 1d**	**1P + 4d**	**2P + 1d**	**2P + 4d**	**1P + 1d**	**1P + 4d**	**2P + 1d**	**2P + 4d**	**1P + 1d**	**1P + 4d**	**2P + 1d**	**2P + 4d**	**1P + 1d**	**1P + 4d**	**2P + 1d**	**2P + 4d**

	**Net photosynthesis (Pn)**															

Median	2.10	−1.12	0.54	0.35	1.56	2.52*	0.81	1.31	2.32	2.39	2.31	0.95	3.84	3.03	3.33	3.58*
SD	0.58	0.90	0.09	0.73	0.25	0.91	0.96	0.36	1.20	0.70	0.11	0.44	2.25	2.11	2.09	2.12

	**Intercellular CO_2_ (Ci)**															

Median	324.4	482.3	354.9	375.8	278.6	170.2*	364.8	254.9*	321.5	313.3	253.6	359.8	296.4	311.6	310.2	290.8*
SD	17.90	255.61	2.65	29.5	18.4	115.2	21.9	30.6	17.54	33.7	41.9	9.9	176.6	189.7	188.4	170.4

Data are the medians of three values ± SD. The asterisk (*) indicates a significant difference (Mann-Whitney U test, p < 0.05) between treated (T) and non-treated (NT) conditions at the same kinetic point. 1P = first post infection treatment; 2P = second post infection treatment.

In Assay 2 cultivars appeared to respond differently to the LC2017 treatments (Table 3). For “Chardonnay,” a significant and strong decrease of Pn and G_s_ was observed at 2B + 4d in both NT and T that suggested a non-stomatal limitation, as observed after the two treatments (P1 and P2) in Assay 1. For “Cabernet sauvignon,” its G_s_ also decreased across the time-points, but especially at 2B + 4d. Similarly, but except at 2B + 1d, the LC2017-treated vines featured lower Pn values when compared with NT. Unlike in Assay 1, no remarkable modifications of photosynthetic parameters were observed following the *Botryosphaeriaceae* challenge (Table 6). Details for the other recorded photosynthetic parameters in Assay 2 are also available in Supplementary Tables 6, 7.

**TABLE 6:** Photosynthetic parameters (Pn, Gs, Ci, and Tr) recorded during Assay 2 one (+ 1 d) and four (+ 4 d) days after the treatments on non-inoculated grapevines “Chardonnay” and “Cabernet sauvignon.”

	Chardonnay Assay 2				Cabernet sauvignon Assay 2			
	Ds 99-7 NT		Ds 99-7 T		Ds 99-7 NT		Ds 99-7 T	
	3P + 1d	3P + 4d	3P + 1d	3P + 4d	3P + 1d	3P + 4d	3P + 1d	3P + 4d
	**Net Photosynthesis (Pn)**							

Median	6.23	7.27	7.31	7.27	3.37	4.72	5.36*	4.64
SD	0.70	2.16	1.68	2.16	0.75	0.39	1.07	0.90

	**Intercellular CO_2_ (Ci)**							

Median	302.7	271.1	304.3	310.7	251.0	185. 8	209. 9	145.2*
SD	8.3	21.4	2.1	11.5	17.2	23.6	10.8	58.5

	**Chardonnay Assay 2**				**Cabernet sauvignon Assay 2**			
	**Np bt67 NT**		**Np bt67 T**		**Np bt67 NT**		**Np bt67 T**	
	**3P + 1d**	**3P + 4d**	**3P + 1d**	**3P + 4d**	**3P + 1d**	**3P + 4d**	**3P + 1d**	**3P + 4d**

	**Net Photosynthesis (Pn)**							

Median	4.27	3.88	5.08	4.85	1.25	2.30	6.32*	5.49
SD	1.49	0.88	0.99	1.40	0.08	0.84	1.90	2.60

	**Intercellular CO_2_ (Ci)**							

Median	294.3	292.7	286.0	319.0	253.2	243.2	170.1*	195.2
SD	11.7	14.7	35.8	25.5	51.8	40.6	13.8	75.2

Data are the medians of three values ± SD. The asterisk (*) indicates a significant difference (Mann-Whitney U test, p < 0.05) among the four time-point values within the same condition [T (treated) or NT (non-treated)]. 2B = second treatment before infection; 3P = post-infection treatment.

#### Plant Fresh Weight

**FIGURE 5:**
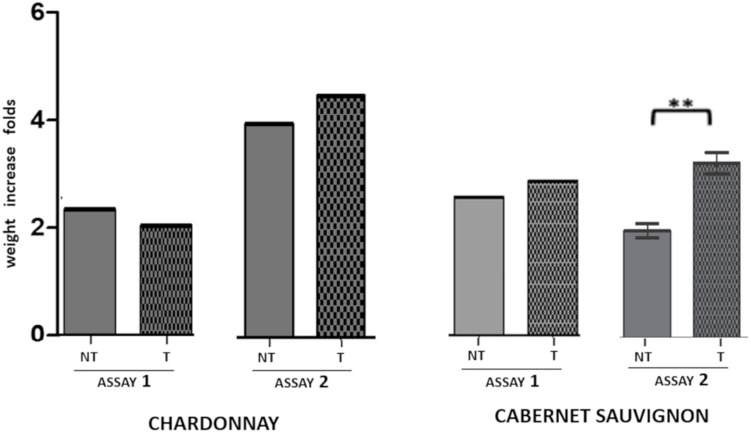
Effect of LC2017 treatments on the vine fresh weight, expressed as weight increase folds recorded at the end of each assay (60 dpi) independently from kind and pathogens presence/absence. On the left, the effect of LC2017 treatments on cultivar “Chardonnay”; on the right the effect on “Cabernet sauvignon.” The symbol ^∗∗^ indicates statistical difference for *p* < 0.01. T = LC2017 treated NT = not treated.

The effect of LC2017 on the whole plant fresh weight recorded in the study is presented in Figure 5. Pooling all the data in the two groups T and NT, LC2017-treated vines showed a different behavior according to the considered treatment strategy (Assay 1 or Assay 2) and cultivars. Overall, no negative effects of LC2017 on plant development were observed. On “Cabernet sauvignon,” LC2017 induced an increase in whole plant fresh weight in both the assays, significant in Assay 2. On the contrary, no significant effect was recorded on “Chardonnay.” A detailed analysis on plant growth is showed in Supplementary Figure 1.

### Effect of LC2017 Treatments on *Botryosphaeriaceae* Pathogens *in vitro* and *in planta*

#### *In vitro* Effect of LC2017 Against *Botryosphaeriaceae*

**FIGURE 6:**
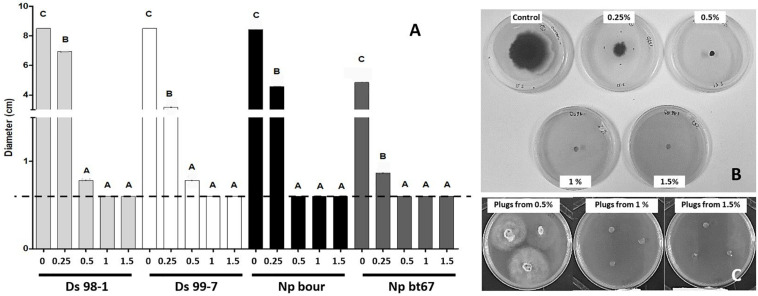
Direct effect of LC2017 on pathogens development *in vitro* observed at 9 dpi. In **(A)**, the result of the different concentrations on the colony growth of the four *Botryosphaeriaceae* used in this study. To same letters correspond no statistical difference. The dotted line represents the initial diameter of mycelial plugs. In **(B)**, the development of *D. seriata* strain Ds 98-1 at 4dpi in plates enriched with LC2017 at different concentrations. In **(C)**, the behavior of Ds 98-1 plugs after 3 days in non-enriched PDA plates: growth recovery from LC2017 at 0.5% (LC2017 fungistatic effect) no growth from LC2017 at 1 and 1.5% (LC2017 fungicide effect).

At 9 dpi, LC2017 significantly inhibited fungal growth, but at varying intensities that depended on both the LC2017 concentration and pathogen species (Figure 6A). At the lowest concentration (0.25% v/v), inhibition by LC2017 was limited but statistically significant for all four strains. At 0.5%, LC2017’s fungal inhibition reached a near maximum, with no significant differences found among species, nor with the effects of LC2017 when applied at 1% and 1.5% which prevented the growth of all tested strains (Figure 6A). To distinguish a potential fungicidal effect from a fungistatic one, mycelial plugs that had stunted or no growth after 9 dpi (Figure 6B) were transferred onto PDA plates deprived of LC2017. Almost all mycelia started to grow again, except for the *D. seriata* 98-1 mycelia previously exposed to LC2017 at 1% and 1.5% (Figure 6C).

The *in vitro* tests thus highlighted the potent fungistatic property of LC2017 on both *Botryosphaeriaceae* species, when applied at a low concentration (0.25%). Additionally, LC2017 may also exert fungicidal effects on *D. seriata* 98-1 when applied at 1% and 1.5%.

### Effect of LC2017 on Botryosphaeria Dieback Development *in planta*

**FIGURE 7:**
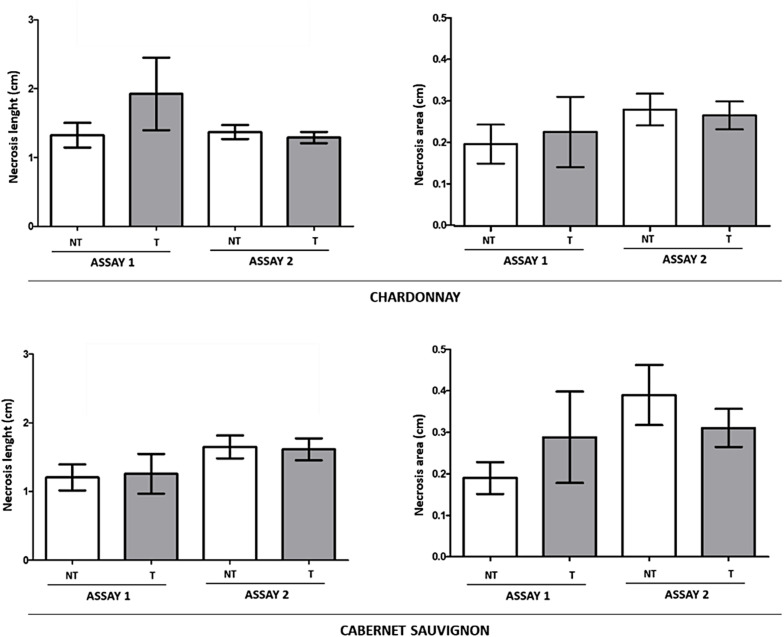
Global effect of LC2017 treatments on internal necrosis length and area development recorded in Assay 1 and Assay 2 in rooted cuttings “Chardonnay” **(upper part)** and “Cabernet sauvignon” **(lower part)** when inoculated with the two *Botryosphaeriaceae* species *D. seriata* and N. parvum and recorded at 60 dpi (W2 in Figure 1).

No symptoms of necrosis were observed in vines inoculated with sterile PDA plugs (*data not shown*) and no pathogens were re-isolated (Table 7). In stark contrast, internal necrosis lengths and necrosis areas were observed at 60 dpi in all *Botryosphaeria*-inoculated cuttings of the two grapevine cultivars (Figure 7), whether sprayed with LC2017 (T) or not (NT), and pathogens were recovered from the inside, except for *D. seriata* Ds 99-7 that went undetected in the LC2017-treated “Cabernet sauvignon” in Assay 1 (Table 7).

**TABLE 7:** Re-isolation percentages of the four Botryosphaeria-dieback pathogens inoculated in vines “Chardonnay” and “Cabernet sauvignon” treated with LC2017 (T) compared to non- treated (NT) in Assay 1 and 2 at 60 dpi.

	Assay 1				Assay 2			
	Chardonnay		Cabernet sauvignon		Chardonnay		Cabernet sauvignon	
	NT	T	NT	T	NT	T	NT	T
Control	0.0%	0.0%	0.0%	0.0%	0.0%	0.0%	0.0%	0.0%
Ds 98-1	35.1%	37.5%	27.5%	32.5%	23.8%	85.7%	23.8%	33.3%
Ds 99-7	82.5%	2.6%	8.6%	0.0%	38.1%	33.3%	57.1%	23.8%
Np bour	85.4%	35.1%	43.8%	60.0%	57.1%	95.2%	42.9%	61.9%
Np bt67	62.5%	90.3%	70.8%	68.8%	100.0%	85.7%	33.3%	42.9%

In both Assay 1 and 2, irrespective of the LC2017 spray timing, no significant differences emerged in the recorded necrosis length between non-treated (NT) and LC2017-treated (T) plants, in both “Chardonnay” and “Cabernet sauvignon,” even though the necrosis in LC2017-treated vines were lengthier than in the corresponding untreated plants in Assay 1 (Figure 7). Interestingly, our *in planta* tests confirmed the disparate aggressiveness between the two *Botryosphaeriaceae* species, in that *N. parvum* was more virulent than *D. seriata* when infecting *V. vinifera* (Supplementary Figure 2), consistent with reports by several authors (Úrbez-Torres and Gubler, 2009; Pitt et al., 2013).

## Discussion

In this study, our objective was to assess the efficiency of LC2017, a formulated product innovative for its low copper content and delivery system, as elicitor of genes related to grapevine defense system and possible plant protection product (PPP) against the Botryosphaeria dieback disease. In so doing, we also evaluated its impact on plant growth and photosynthetic activity.

### LC2017 Elicits the Defenses of Grapevine Cuttings

This study design allowed us to ascertain the elicitation ability of LC2017 toward several genes known to be related to plant defenses (Figures 4–6). In short, our study ranked the elicitation potential of LC2017 at the same level as that of BTH, a commonly marketed elicitor. Among the genes elicited and included in our RT-qPCR approach, were those related to the synthesis of chitinase and glucanase, and to the biosynthesis pathways (*PAL*, *STS*) of some phenolic compounds that exert a role in grapevine tolerance to biotic stress (i.e., pathogens, insects, herbivore attacks) and abiotic ones (UV-light) (Chong et al., 2009; Trotel-Aziz et al., 2019). Upon the *Botryosphaeriaceae* infections, the LC2017 treatments significantly increased the plant defenses in comparison with the NT conditions. Similar results were presented in the recent paper Battiston et al. (2021) with *in planta* assay against *Phaeoacremonium minimum*, a causal agent in Esca disease, another relevant GTDs.

In the meantime, genes related to the “arsenite-recovery” effect were also up-regulated by the LC2017 treatments, especially in Assay 1, under the post infection treatment strategy. The “arsenite-recovery” genes were chosen based on the findings of a comparative transcriptomic study of healthy (naturally or resilient) and GTD-infected vines treated or not with arsenite (Vallet et al., 2017). Arsenite was the only PPP available at the beginning of the last decade for controlling GTDs, but it has been recently banned (Mondello et al., 2018b). As observed for the Esca complex disease (Vallet et al., 2017), several genes altered in their expression levels in GTD-symptomatic plants may resume the same expression level as healthy counterparts when the GTD-symptomatic plants are treated with sodium arsenite. These authors indicated that sodium arsenite’s protective effect might result from how it affects host-plant gene expression to trigger resiliency. Taken together, our results suggest that LC2017 may mimic both an elicitor-like effect (i.e., BTH) as well as an “arsenite-recovery” effect, both of which would be useful for vines to better resist infection or recover from GTD symptoms.

Additionally, it appears that LC2017 was able to have this elicitor effect without limiting environmental damages (because of its low copper content) or phytotoxicity. Indeed, LC2017-treated vines did not exhibit any negative impact on photosynthesis nor chlorophyll metabolism (Tables 3–6), unlike Cu-treated vines (Leng et al., 2015), despite having similar defense gene induction profiles.

Finally, our study also allowed the analysis of the independent effect of fungal pathogens on both plant defense responses and cultivar susceptibility. We first confirmed the higher susceptibility of “Cabernet sauvignon” toward GTDs when compared with “Chardonnay.” When infected by *D. seriata* or *N. parvum*, only a few host-plant defense genes were significantly induced in “Cabernet sauvignon” compared to “Chardonnay.” Recently, Wu et al. (2020) and Guerin-Dubrana et al. (2019) also reported this various susceptibility of the two cultivars to GTD pathogens.

### LC2017 Harmless for Grapevine Cuttings Physiology

As already reported by Battiston et al. (2018), no copper-related phytotoxic effects were visually observed in LC2017-treated plants. The copper concentration is low in LC2017 (35 g/L), far below that of other copper-based PPPs described as being phytotoxic for grapevine (Apel and Hirt, 2004; Yruela, 2005), even compared to those with innovative formulations (Dagostin et al., 2011). In agreement with the absence of toxicity signs on grapevine leaves, treating them with LC2017 did not appear to adversely affect the growth of either “Chardonnay” or “Cabernet sauvignon” (Figure 5). In fact, some evidence for growth-promoting effects was even observed in the LC2017-treated plants, especially in the presence of colonizing Botryosphaeria-dieback pathogens. On this point, the behavior of LC2017 seems to diverge from that characterizing the “classic” fungicides (Ahmed et al., 1983; Saladin et al., 2003). Growth promotion could be associated with the observed boost of photosynthetic activity as revealed by a better CO_2_ assimilation rate (i.e., low Ci values, see Table 3), even if not confirmed by changes in the expression of both targeted photosynthesis-related genes (Table 2). This hypothesis is also supported by the overall stability of other photosynthetic parameters, namely G_s_ and Tr, in all the experimental conditions. Generally, during fungicide treatments (especially with copper- and sulfur-based pesticides) the photosynthetic parameters values decrease, indicating a changed photosynthetic efficiency that finally determines the assimilation of carbon in the plant (Petit et al., 2012). Importantly, in our study, no differences were observed in the two assays.

### *In vitro* LC2017 Fungistatic Effect Against Botryosphaeria Dieback Pathogens

LC2017 exerted a marked fungistatic effect at 0.5% (175 μg/mL of Cu^2+^) against four *Botryosphaeriaceae* strains (Figure 6) as observed against *P. minimum* (Battiston et al., 2021). These results confirm the concentration range sensibility of *Botryosphaeriaceae* to copper, as observed *in vitro*, by Bester et al. (2007) with copper ammonium acetate (ineffective below 20 μg/mL) and by Amponsah et al. (2012) with copper hydroxide (EC_50_ 84 μg/mL). Regarding its feasibility use as a PPP, the LC2017’s fungistasis should be considered positively, especially in light of the possible emergence of pathogens that have evolved resistance to fungicides, as already observed for other grapevine diseases, such as downy mildew (*Erysiphe necator*) and gray mold (*Botrytis cinerea*), to name a few (Gessler et al., 2011; Hauschildt et al., 2020). Furthermore, fungistasis *via* LC2017 may preserve the vine’s fungal microbiome, which usually hosts several plant growth-promoting and beneficial microorganisms (Pinto et al., 2014; Pinto and Gomes, 2016).

### Greenhouse Protective Effect of LC2017 on Infected Cuttings

The above-described LC2017 behavior was further confirmed with the *in planta* tests. *In planta*, upon Assays 1 and 2 control trials, pathogens were re-isolated and recovered from the imposed T and NT conditions at 60 dpi (Table 7), but this revealed no influence of the spray timing strategy toward the pathogens’ living capacity. Although the *in planta* assays do not permit us to highlight any LC2017 effect on pathogens, the reduction in stem necrosis lengths in the plants pre-treated with LC2017 in Assay 2, even if not significant, does highlight the benefit of a preventive induction impact beyond pursuing a post infection control strategy. We hypothesize that this could arise from the fungistatic capacity of LC2017. As a future PPP, such a fungistatic effect can be considered an asset for LC2017 *in planta*, by allotting to a plant more time to maintain its fitness and to develop robust defenses (Mondello et al., 2018b; Trotel-Aziz et al., 2019). With a stronger elicitation of some plant defense responses, this strategy might limit grapevine colonization by pathogens. Finally, our measurements of internal necrosis also confirmed the greater and well-documented aggressiveness of *N. parvum* as compared to *D. seriata* (Úrbez-Torres and Gubler, 2009; Pitt et al., 2013).

### LC2017’s Potential as a PPP for Controlling GTDs in the Field

Despite being carried out under controlled condition, our study nonetheless highlights several interesting aspects of LC2017 treatments in view of their potential use in vineyards. Its low copper content and innovative delivery system (Battiston, 2018; Battiston et al., 2018, 2019, 2021) can improve the AI’s efficiency, thereby respecting limitation in copper delivery while simultaneously enabling the control of different fungal and bacterial diseases. Beside the well-known copper fungicide effects, LC2017 acted much like BTH against downy and powdery mildew through its elicitor effect. According to Dufour et al. (2013), the efficiency of BTH to control these diseases is strongly associated with up-regulation of key host-plant defense genes, such as those encoding for pathogenesis-related (PR) proteins and those repressed by the pathogen.

Unlike most fungicides, LC2017 stimulates plant growth and photosynthesis, and elicits several genes linked to plant defenses, which collectively could play a decisive role in blocking/limiting infection in natural settings for those hard-controlling diseases like GTDs are. Rego et al. (2014) were the first to record this positive synergy between copper and defense genes’ elicitation in Portugal’s vineyard. They observed that the use of a copper-based product (Cuprocol) coupled with BTH treatments reduced both the Botryosphaeria dieback incidence and severity in the field tests. Since elicitation alone cannot be considered as a “miracle-key” to control plant diseases (Delaunois et al., 2014), it appears that the “right choice” is to combine in a PPP both the traditional mechanisms (copper) and the new ones (gene elicitation) to achieve the best GTD control, in particular for those PPPs having an AI whose direct effects on pathogens are not so selective. To validate this hypothesis and to complete the experimental studies under controlled conditions (Battiston et al., 2021, and this study), trials in vineyard are ongoing. The expected epidemiologic and plant responses results and the impact on the plant microbiome could give a more precise evaluation on the possible use of LC2017 for the control of GTDs in natural condition.

## Data Availability Statement

The original contributions presented in the study are included in the article/Supplementary Material, further inquiries can be directed to the corresponding authors.

## Author Contributions

VM performed all the experiments, analyzed transcriptomic, physiological and disease development data, and wrote the manuscript. OF and PT-A critically revised the manuscript, transcriptomic analyses, and statistics. J-FG Guise supervised greenhouse experiments. FF set-up the experimental design, supervised the study, and critically revised the manuscript. All authors contributed to the article and approved the submitted version.

## Conflict of Interest

The authors declare that the research was conducted in the absence of any commercial or financial relationships that could be construed as a potential conflict of interest. The reviewer MM-R declared a past co-authorship with one of the author FF to the handling editor.

## Publisher’s Note

All claims expressed in this article are solely those of the authors and do not necessarily represent those of their affiliated organizations, or those of the publisher, the editors and the reviewers. Any product that may be evaluated in this article, or claim that may be made by its manufacturer, is not guaranteed or endorsed by the publisher.

## Footnotes

1
http://imagej.nih.gov/ij

2
www.graphpad.com

## Supplementary Material

Supplementary Figure 1 Treatments performed in greenhouse on vegetative cuttings. In **(A**) leaves of “Chardonnay” treated with LC2017 [at 0.5%. In **(B)** the assay 2 first LC2017 treatment (1B) of on rooting cuttings not in vegetation, previously cut in the upper part **(C)**] to mimic the winter pruning wounds in vineyard.

Supplementary Figure 2 Effect of LC2017 treatments on the whole vine fresh weight, expressed as weight increase folds and recorded at the end of each test (60 dpi). In the upper part, the effect of LC2017 treatments for each “Chardonnay” condition in Assay 1 **(A)** and Assay 2 **(B)**; in the lower part, the effect of LC2017 treatments for each “Cabernet sauvignon” condition in Assay 1 **(C)** and Assay 2 **(D)**. The symbol ^∗^ indicates statistical difference for *p* < 0.05; ^∗∗^ indicates statistical difference for *p* < 0.01. T = LC2017 treated NT = not treated.

Supplementary Figure 3 Internal necrosis length and area development in rooted cuttings “Chardonnay” **(A,B)** and “Cabernet sauvignon” **(C,D)** inoculated with *D. seriata* and *N. parvum* strains at 60 dpi. *Evaluation of LC2017 treatment*: to different letters correspond statistical differences (*p* > 0.05) between treatments (NT – T) of the same condition and year. In Assay 1, treatments with LC2017 on inoculated vines often induced longer and larger necrosis in both cultivars compared to NT, with statistical significances found only for necrosis area in Np bt67-inoculated “Cabernet sauvignon.” In Assay 2, LC2017-treated and inoculated vines showed shorter and smaller necrosis compared to the NT ones, even if with no significance. *Evaluation of pathogen aggressiveness*: to different symbols correspond statistical differences (*p* > 0.05) in necrosis length/area produced by the four tested strains, as recorded in the same year (^∗^ for not treated, □ for LC2017). Globally, *N. parvum* induced longer necrosis in both assays compared to *D. seriata*.
