# Supplementary figures and images for: In planta Activity of the Novel Copper Product HA + Cu(II) Based on a Biocompatible Drug Delivery System on Vine Physiology and Trials for the Control of Botryosphaeria Dieback
Source: Front Plant Sci. 2021 Sep 3;12:693995. doi: 10.3389/fpls.2021.693995 (PMC8446610; doi:10.3389/fpls.2021.693995)

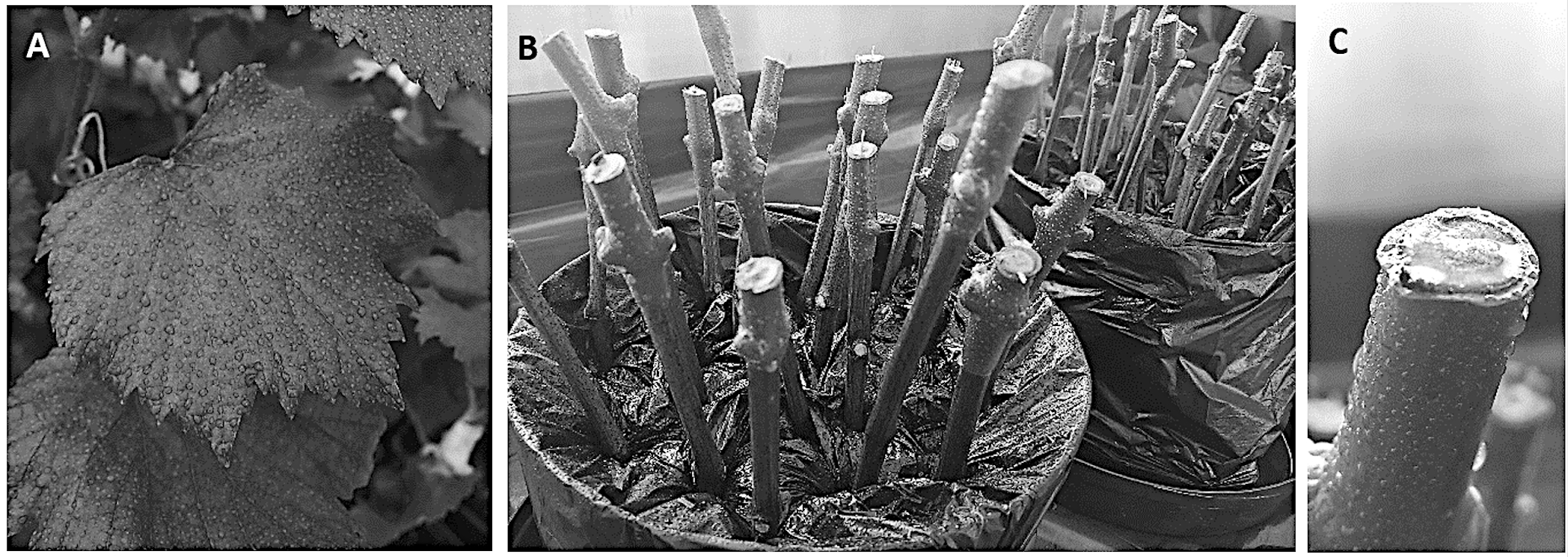

Supplement: Supplementary Figure 1 — Treatments performed in greenhouse on vegetative cuttings. In (A) leaves of “Chardonnay” treated with LC2017 [at 0.5%. In (B) the assay 2 first LC2017 treatment (1B) of on rooting cuttings not in vegetation, previously cut in the upper part (C)] to mimic the winter pruning wounds in vineyard. [file Image_1.JPEG]

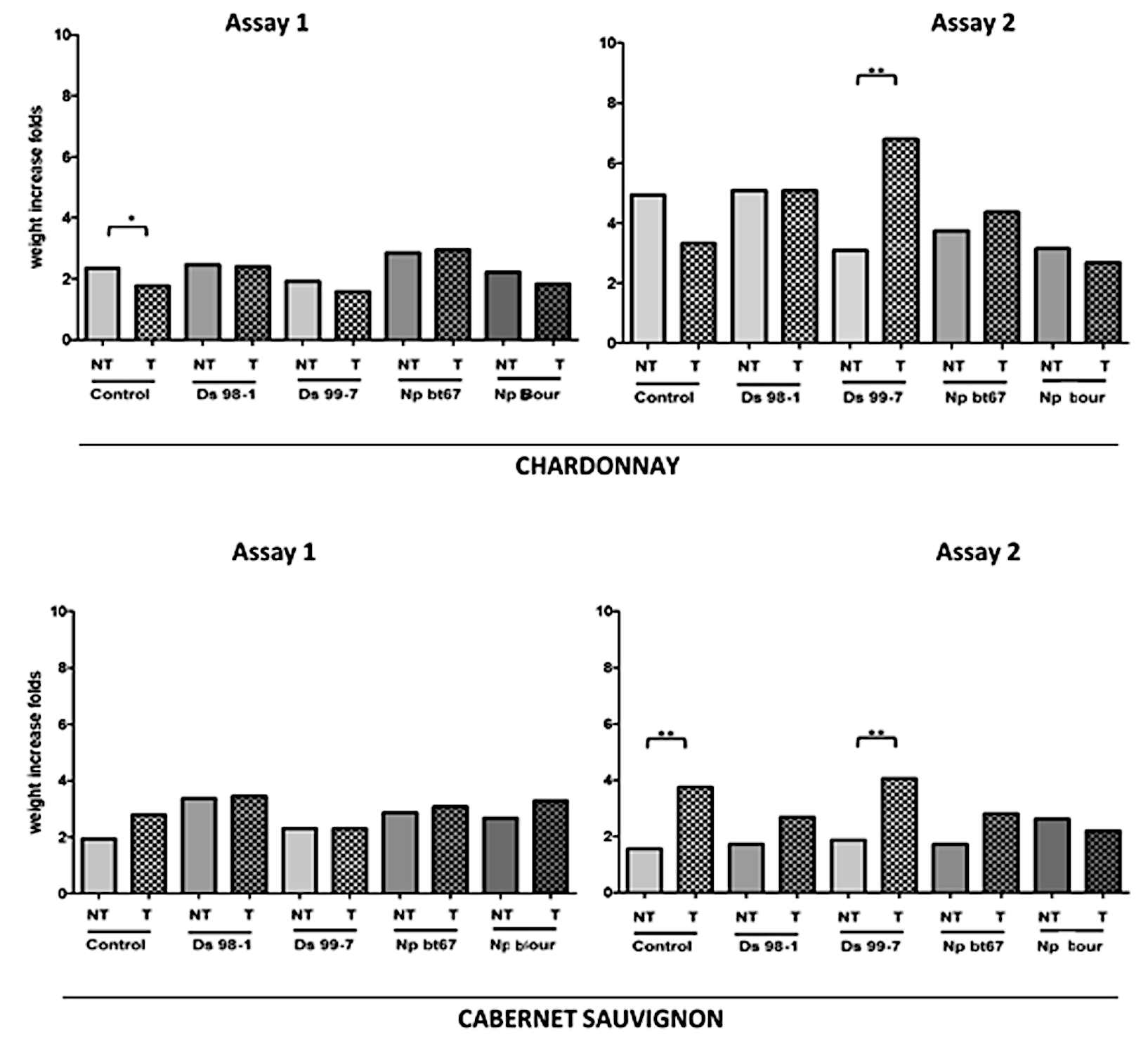

Supplement: Supplementary Figure 2 — Effect of LC2017 treatments on the whole vine fresh weight, expressed as weight increase folds and recorded at the end of each test (60 dpi). In the upper part, the effect of LC2017 treatments for each “Chardonnay” condition in Assay 1 (A) and Assay 2 (B); in the lower part, the effect of LC2017 treatments for each “Cabernet sauvignon” condition in Assay 1 (C) and Assay 2 (D). The symbol ∗ indicates statistical difference for p < 0.05; ∗∗ indicates statistical difference for p < 0.01. T = LC2017 treated NT = not treated. [file Image_2.JPEG]

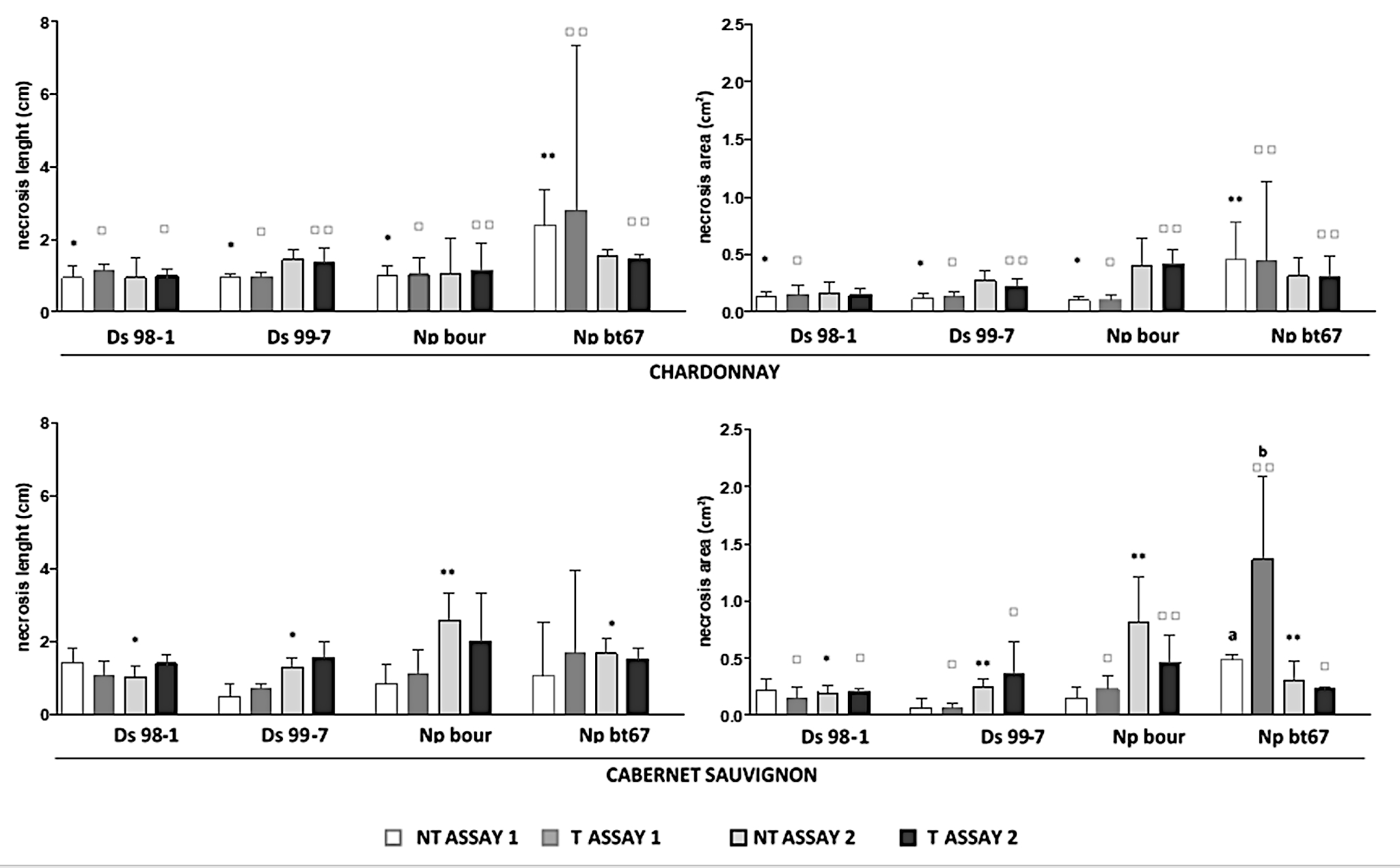

Supplement: Supplementary Figure 3 — Internal necrosis length and area development in rooted cuttings “Chardonnay” (A,B) and “Cabernet sauvignon” (C,D) inoculated with D. seriata and N. parvum strains at 60 dpi. Evaluation of LC2017 treatment: to different letters correspond statistical differences (p > 0.05) between treatments (NT – T) of the same condition and year. In Assay 1, treatments with LC2017 on inoculated vines often induced longer and larger necrosis in both cultivars compared to NT, with statistical significances found only for necrosis area in Np bt67-inoculated “Cabernet sauvignon.” In Assay 2, LC2017-treated and inoculated vines showed shorter and smaller necrosis compared to the NT ones, even if with no significance. Evaluation of pathogen aggressiveness: to different symbols correspond statistical differences (p > 0.05) in necrosis length/area produced by the four tested strains, as recorded in the same year (∗ for not treated, □ for LC2017). Globally, N. parvum induced longer necrosis in both assays compared to D. seriata. [file Image_3.JPEG]
