# Supplementary material for: In planta Activity of the Novel Copper Product HA + Cu(II) Based on a Biocompatible Drug Delivery System on Vine Physiology and Trials for the Control of Botryosphaeria Dieback
Source: Front Plant Sci. 2021 Sep 3;12:693995. doi: 10.3389/fpls.2021.693995 (PMC8446610; doi:10.3389/fpls.2021.693995)
Supplement: Supplementary file 4 [file Data_Sheet_1.pdf]

Table S1. Primers of genes analyzed by quantitative real-time reverse-transcription polymerase chain reaction.

Function	Gene	Primer Sequences	GenBank or TC TIGR* Accession Number	Reference
Reference genes	*EF1* (EF1-α elongation factor)	5′-GAACTGGGTGCTTGATAGGC-3′<br>5′-AACCAAAATATCCGGAGTAAAAGA-3′	GU585871	Gutha L.R. et al., 2010. BMC Plant Biol. 10, 187
	60SRP (60S ribosomal protein L18)	5′-ATCTACCTCAAGCTCCTAGTC-3′<br>5′-CAATCTTGTCCTCCTTTCCT-3′	XM_002270599	Goremykin VV. et al., 2009. Mol Biol Evol, Jan;26(1):99-110
	39SRP (39S ribosomal protein L41*)*	5′-AACCAAAATATCCGGAGTAAAAGA-3′<br>5′-GACTGACTTCAAGCTTAAACC-3′	XM_002285709	Goremykin VV. et al., 2009. Mol Biol Evol, Jan;26(1):99-110
Phenylpropanoid metabolism	*PAL* (Phenylalanine ammonia lyase)	5′-TCCTCCCGGAAAACAGCTG-3′<br>5′-TCCTCCAAATGCCTCAAATCA-3′	X75967	Sparvoli F. et al., 1994. Plant Mol. Biol. 24 (5), 743-755
	STS (Stilbene synthase)	5′-AGGAAGCAGCATTGAAGGCTC-3′<br>5′-TGCACCAGGCATTTCTACACC-3′	FJ851185	Xu W. et al., 2011 J. Exp. Bot. 62 (8), 2745-2761
Defense protein	*CHIT4C* (Chitinase class V)	5′-TCGAATGGGATGGTGGAAA -3′<br>5′-TCCCCTGTCGAAACACCAAG -3′	NM_001281244	Da Silva C. et al., 2013. Plant Cell 25 (12), 4777-4788
	GLUC (β-1,3 glucanase)	5′-TCAATGGCTGCAATGGTGC-3′<br>5′-CGGTCGATGTTGCGAGATTTA-3′	DQ267748	Romero I. et al, 2006. J Postharvest Biol. Technol. 41 (1), 9-15
	PR1 (pathogenesis-related protein 1)	5′- GGAGTCCATTAGCACTCCTTTG -3′<br>5′- CATAATTCTGGGCGTAGGCAG -3′	XM_002273752	Venturini L. et al., 2013. BMC Genomics, Jan 18;14:41
	PR10 (pathogenesis-related protein 10)	5′- CGTTAAGGGCGGCAAAGAG -3′<br>5′- GCATCAGGGTGTGCCAAGA -3′	DQ396809	Robert N. et al, 2001. European Journal of Plant Pathology 107, 249–261.
Detoxification and Stress tolerance	*GST1* (Glutathione s-transferase 1)	5′- TGCATGGAGGAGGAGTTCGT -3′<br>5′- CAAGGCTATATCCCCATTTTCTTC -3′	NM_001281248	Da Silva C., et al., 2013. Plant Cell 25 (12), 4777-4788
Photosynthesis	*PsbP1 (oxygen-evolving enhancer PsbP subunit of photosystem I)*	5′-TGTCCCCAGCCTGTACCTTG-3′<br>5′-GCTGACGGAGATGAAGGTGG-3′	AY222741	Spagnolo A. et al., 2012. Journal of Proteome Research, 11(1), 461–475.
	RbcL (RuBisCo large sub-unit)	5′-AATTTTTCCTCCACGGCGATA -3′<br>5′-ATCTGCGCCCGCCTTTATA -3′	TC57584	Letousey P. et al., 2010. Phytopathology, 100(5), 424–431.
Arsenite recovery genes	*PME25* (pectin esterase inhibitor)	5′-TGTCGGAGCTGAATGTGGA-3′<br>5′-CGATCCCGCTAAGCAGAGTTT-3′	XM_002273391.4	Fontaine F., *unpublished*
	MSR (Peptide methionine sulfoxide reductase))	5′-GCATTTGGGCGTGTAGATGG-3′<br>5′-GCTTCAGTGTGGCCTGTTCT -3′	XM_010662104.2	Fontaine F., *unpublished*
	WRKY (transcription factors)	5′-GGGCAGAAGGACATTCTTGGA -3′<br>5′-AGGGATCTTCATCCGAACGC -3′	XM_002272684.3	Fontaine F., *unpublished*
	Hyd2 (ABA 8' hydroxylase 2)	5′-GAAGCTTGTTCTCCGAGCCT -3′<br>5′-TATTGATCGACCGGCCTTCC -3′	NM_001281052.1	Fontaine F., *unpublished*

* see http://www.jcvi.org/cms/research/projects/tdb/overview/

Table S2. Kinetics of stomatal conductance (Gs) and transpiration rate (Tr) values recorded during Assay 1 one (+1d) and four (+4d) days after 1P and 2P treatments vines of 'Chardonnay' and 'Cabernet sauvignon' artificially inoculated with *Diplodia seriata* strain Ds 98-1 in Assay 1. Data reported are medians of 3 values ± SD. The asterisk (*) indicates a statistical difference (Mann-Whitney U test, $p<0.05$) between treated (T) and non-treated (NT) conditions at the same kinetic point. 1P = first treatment post inoculation, 2P = second treatment post inoculation.

	CHARDONNAY – ASSAY 1								CABERNET SAUVIGNON – ASSAY 1							
	Ds98-1 NT				Ds 98-1 T				Ds98-1 NT				Ds 98-1 T			
	1P+1d	1P+4d	2P+1d	2P+4d	1P+1d	1P+4d	2P+1d	2P+4d	1P+1d	1P+4d	2P+1d	2P+4d	1P+1d	1P+4d	2P+1d	2P+4d
	Stomatal conductance (Gs)															
**Median**	0.16	0.09	0.02	0.02	0.13*	0.13	0.12*	0.04	0.10	0.04	0.02	0.02	0.03	0.03	0.07	0.02
**SD**	0.02	0.04	0.04	0.05	0.01	0.05	0.04	0.01	0.06	0.03	0.03	0.01	0.06	0.07	0.05	0.03
	Transpiration rate (Tr)															
**Median**	2.35	1.63	0.42	0.30	1.99*	2.37	1.86	0.65	1.77	0.69	0.28	0.33	0.56	0.52	1.03	0.34
**SD**	0.12	0.71	0.61	0.81	0.16	1.01	0.59	0.25	0.99	0.49	0.43	0.20	0.97	1.18	0.70	0.64

Table S3. Kinetics of stomatal conductance (Gs) and transpiration rate (Tr) values recorded during Assay 1 one (+1d) and four (+4d) days after 1P and 2P treatments vines of 'Chardonnay' and 'Cabernet sauvignon' artificially inoculated with *Diplodia seriata* strain Ds 99-7. Data reported are medians of 3 values ± SD. The asterisk (*) indicates a statistical difference (Mann-Whitney U test, $p<0.05$) between treated (T) and non-treated (NT) conditions at the same kinetic point. 1P = first treatment post inoculation, 2P = second treatment post inoculation.

	CHARDONNAY – ASSAY 1								CABERNET SAUVIGNON – ASSAY 1							
	Ds 99-7 NT				Ds 99-7 T				Ds 99-7 NT				Ds 99-7 T			
	1P+1d	1P+4d	2P+1d	2P+4d	1P+1d	1P+4d	2P+1d	2P+4d	1P+1d	1P+4d	2P+1d	2P+4d	1P+1d	1P+4d	2P+1d	2P+4d
	Stomatal conductance (Gs)															
**Median**	0.03	0.01	0.02	0.03	0.03	0.02	0.04	0.02	0.06	0.02	0.02	0.03	0.04	0.03	0.04	0.02
**SD**	0.00	0.06	0.02	0.01	0.04	0.02	0.11	0.04	0.02	0.01	0.02	0.01	0.01	0.01	0.00	0.01
	Transpiration rate (Tr)															
**Median**	0.54	0.21	0.46	0.58	0.55	0.26	0.72	0.32	0.97	0.41	0.29	0.44	0.60	0.53	0.73	0.38
**SD**	0.07	1.04	0.39	0.23	0.53	0.34	1.64	0.67	0.19	0.24	0.29	0.27	0.24	0.29	0.10	0.24

Table S4. Kinetics of stomatal conductance (Gs) and transpiration rate (Tr) values recorded during Assay 1 one (+1d) and four (+4d) days after each curative treatment vines of 'Chardonnay' and 'Cabernet sauvignon' artificially inoculated with *Neofusicoccum parvum* strain Np bour. Data reported are medians of 3 values ± SD. The asterisk (*) indicates a statistical difference (Mann-Whitney U test, $p<0.05$) between treated (T) and non-treated (NT) conditions at the same kinetic point. 1P = first treatment post inoculation, 2P = second treatment post inoculation.

	CHARDONNAY – ASSAY 1								CABERNET SAUVIGNON – ASSAY 1							
	Np bour NT				Np bour T				Np bour NT				Np bour T			
	1P+1d	1P+4d	2P+1d	2P+4d	1P+1d	1P+4d	2P+1d	2P+4d	1P+1d	1P+4d	2P+1d	2P+4d	1P+1d	1P+4d	2P+1d	2P+4d
	Stomatal conductance (Gs)															
**Median**	0.05	0.07	0.07	0.07	0.04	0.03	0.03	0.03	0.10	0.08	0.04	0.07	0.07	0.06	0.05	0.05
**SD**	0.16	0.08	0.04	0.12	0.04	0.10	0.04	0.01	0.05	0.03	0.02	0.03	0.01	0.02	0.02	0.01
	Transpiration rate (Tr)															
**Median**	0.82	1.13	1.13	1.11	0.76	0.61	0.58	0.35	1.75	1.28	0.64	1.15	1.18	1.08	0.92	0.90
**SD**	2.00	1.31	0.60	1.75	0.66	1.49	0.71	0.23	0.84	0.34	0.39	0.56	0.13	0.25	0.34	0.21

Table S5. Kinetics of stomatal conductance (Gs) and transpiration rate (Tr) values recorded during Assay 1 one (+1d) and four (+4d) days after each treatment in vines of ‘Chardonnay’ and ‘Cabernet sauvignon’ artificially inoculated with *Neofusicoccum parvum* strain Np bt67. Data reported are medians of 3 values ± SD. The asterisk (*) indicates a statistical difference (Mann-Whitney U test, $p<0.05$) between treated (T) and non-treated (NT) conditions at the same kinetic point. 1P = first treatment post inoculation, 2P = second treatment post inoculation.

	**CHARDONNAY – ASSAY 1**								**CABERNET SAUVIGNON – ASSAY 1**							
	**Np bt67 NT**				**Np bt67 T**				**Np bt67 NT**				**Np bt67 T**			
	**1P+1d**	**1P+4d**	**2P+1d**	**2P+4d**	**1P+1d**	**1P+4d**	**2P+1d**	**2P+4d**	**1P+1d**	**1P+4d**	**2P+1d**	**2P+4d**	**1P+1d**	**1P+4d**	**2P+1d**	**2P+4d**
	**Stomatal conductance (Gs)**															
**Median**	0.05	0.02	0.03	0.04	0.02	0.02	0.04	0.01	0.07	0.05	0.03	0.04	0.07	0.06	0.07	0.06
**SD**	0.02	0.02	0.01	0.01	0.03	0.02	0.06	0.01	0.02	0.03	0.01	0.01	0.05	0.08	0.07	0.04
	**Transpiration rate (Tr)**															
**Median**	0.81	0.29	0.46	0.64	0.38	0.36	0.62	0.22	1.43	0.88	0.45	0.78	1.23	1.03	1.23	1.21
**SD**	0.37	0.42	0.10	0.18	0.37	0.42	0.96	0.17	0.33	0.42	0.13	0.22	0.82	1.21	0.98	0.70

Table S6. Net photosynthesis (Pn) and intercellular CO_2 (Ci) values recorded during Assay 2 one (+1d) and four (+4d) days after the post inoculation treatment (3P) on vines of ‘Chardonnay’ and ‘Cabernet sauvignon’ artificially inoculated with *Diplodia seriata* strain Ds 99-7 in 2019. Data reported are medians of 3 values ± SD. The asterisk (*) indicates a statistical difference (Mann-Whitney U test, $p<0.05$) between treated (T) and non-treated (NT) conditions at the same kinetic point. 3P = post infection treatment.

	CHARDONNAY ASSAY 2				CABERNET SAUVIGNON ASSAY 2			
	Ds 99-7 NT		Ds 99-7 T		Ds 99-7 NT		Ds 99-7 T	
	3P+1d	3P+4d	3P+1d	3P+4d	3P+1d	3P+4d	3P+1d	3P+4d
	Stomatal conductance (Gs)							
**Median**	0.12	0.16	0.13	0.16	0.04	0.03	0.05	0.03
**SD**	0.02	0.03	0.04	0.03	0.01	0.02	0.01	0.02
	Transpiration rate (Tr)							
**Median**	1.96	2.41	2.04	2.40	0.74	0.71	0.93	0.53
**SD**	0.21	0.36	0.51	0.36	0.14	0.28	0.17	0.28

Table S7. Net photosynthesis (Pn) and intercellular CO_2 (Ci) values recorded during Assay 2 one (+1d) and four (+4d) days after the post inoculation treatment (3P) on vines of 'Chardonnay' and 'Cabernet sauvignon' artificially inoculated with *Neofusicoccum parvum* strain Np bt67. Data reported are medians of 3 values ± SD. The asterisk (*) indicates a statistical difference (Mann-Whitney U test, $p<0.05$) between treated (T) and non-treated (NT) conditions at the same kinetic point. 3P = post infection treatment

	CHARDONNAY ASSAY 2				CABERNET SAUVIGNON ASSAY 2			
	Np bt67 NT		Np bt67 T		Np bt67 NT		Np bt67 T	
	3P+1d	3P+4d	3P+1d	3P+4d	3P+1d	3P+4d	3P+1d	3P+4d
	Stomatal conductance (Gs)							
**Median**	0.08	0.08	0.06	0.12	0.01	0.03	0.05	0.04
**SD**	0.02	0.02	0.06	0.09	0.01	0.01	0.01	0.02
	Transpiration rate (Tr)							
**Median**	1.31	1.36	1.04	1.95	0.27	0.54	0.95	0.70
**SD**	0.30	0.30	0.87	1.18	0.12	0.25	0.23	0.40
